# Indoleamine 2,3-dioxygenase expression regulates the survival and proliferation of *Fusobacterium nucleatum* in THP-1-derived macrophages

**DOI:** 10.1038/s41419-018-0389-0

**Published:** 2018-03-02

**Authors:** Ying Xue, Han Xiao, Songhe Guo, Banglao Xu, Yuehua Liao, Yixian Wu, Ge Zhang

**Affiliations:** 1 School of Public Health (Shenzhen), Sun Yat-sen University, Guangdong, China; 2 Department of Microbial and Biochemical Pharmacy, School of Pharmaceutical Sciences, Sun Yat-sen University, Guangzhou, China; 3 Department of Clinical Laboratory Medicine, Guangzhou First Municipal People’s Hospital, Guangzhou Medical University, Guangzhou, China

## Abstract

*Fusobacterium nucleatum* (*Fn*) is a tumor-associated obligate anaerobic bacterium, which has a role in the progression of colorectal cancer (CRC). *Fn* can invade and promote colon epithelial cells proliferation. However, how *Fn* survives and proliferates in its host cells remains largely unknown. In this study, we aimed to determine the molecular mechanisms underlying the morphology, survival, and proliferation of *Fn* in THP-1-derived macrophages (dTHP1). For the first time, we found that *Fn* is a facultative intracellular bacterium that can survive and limited proliferate in dTHP1 cells up to 72 h, and a live *Fn* infection can inhibit apoptosis of dTHP1 cells by activating the PI3K and ERK pathways. Both *Fn* bacteria and dTHP1 cells exhibit obvious morphological changes during infection. In addition, Infection of *Fn*-induced indoleamine 2,3-dioxygenase (IDO) expression by TNF-α-dependent and LPS-dependent pathway in a time-dependent and dose-dependent manner, and the IDO-induced low tryptophan and high kynurenine environment inhibited the intracellular multiplication of *Fn* in dTHP1 cells. IDO expression further impaired the function of peripheral blood lymphocytes, permitting the escape of *Fn*-infected macrophages from cell death. IDO inhibition abrogated this effect caused by *Fn* and relieved immune suppression. In conclusion, we identified IDO as an important player mediating intracellular *Fn* proliferation in macrophages, and inhibition of IDO may aggravate infection in *Fn*-associated tumor immunotherapy.

## Introduction

Some aggressive intracellular bacteria can survive and multiply in the cytoplasm of infected macrophages^1^. These facultative intracellular bacteria are shielded from humoral antibodies and can only be eliminated by a cellular immune response^2^. The treatment of intracellular bacteria is an ongoing clinical problem. Currently, numerous studies have confirmed the host–pathogen interaction of some aerobic or facultative anaerobic intracellular bacteria, including *Listeria monocytogenes*, *Legionella pneumophila, Salmonella typhi*, *Mycobacterium tuberculosis*, and *Chlamydia trachomatis*^2^. However, little is known about obligate anaerobic intracellular bacteria and their interaction with host cells.

*Fusobacterium nucleatum* (*F. nucleatum, Fn*) is an opportunistic commensal obligate anaerobic Gram-negative bacterium that is indigenous to the human oral cavity and has a role in periodontal disease. *Fn* has previously been reported to be involved in different infectious processes^3^. Recently, accumulated evidence has demonstrated that *Fn* is associated with the development and carcinogenesis, and promote metastasis in colorectal cancer (CRC)^4–6^. *Fn* can adhere to and invade epithelial cells^7^, and the interaction of *Fn* with CRC cells has been found to promote host cell proliferation^8^. Interestingly, our recent study showed that the overload of *Fn* elicits high levels of *Fn*-specific antibodies in patients with CRC, implying that *Fn* may escape host humoral immune responses by developing inside host cells^9^. Macrophages provide the first line of defense against invading pathogens. Thus, whether *Fn* can survive and multiply in macrophages and its effects on immune functions in host cells need to be explored.

An immunomodulatory role for the enzyme indoleamine 2,3-dioxygenase (IDO), which catalyzes the conversion of tryptophan into kynurenine, has been suggested to have a role in macrophage functions^10^. Increased IDO activity is often associated with tumors and infectious diseases^11^. Several studies have described IDO-dependent T-cell suppression by antigen-presenting cells under many infectious and inflammatory conditions, indicating that biochemical changes due to tryptophan catabolism have a profound effect on T-cell proliferation and effector functions in tissue microenvironments^12,13^.

IDO expression can be induced in macrophages by some bacterial infections^14^. Infection with facultative intracellular bacteria, such as *L. monocytogenes* or *M. tuberculosis*, is associated with IDO induction in various tissues and cell types^15,16^. Interestingly, some obligate intracellular bacteria, such as *C. trachomatis* and *Toxoplasma gondii*, are tryptophan auxotroph. Tryptophan deprivation causes *Chlamydia* to enter a persistent growth^17^. Previous studies have reported that tryptophan is required to stimulate the growth of *Fn*, and *Fn* tryptophanase degrades tryptophan to indole, which can inhibit the growth of Fn in vitro^18^. Furthermore, IDO inhibitors, such as 1-MT (Indoximod), are promising drugs for cancer immunotherapy. Given that a tryptophan-deficient environment caused by IDO in infected macrophages may inhibit the growth of intracellular *Fn*, IDO inhibitors may aggravate infection during *Fn*-associated tumor therapy. To date, live *Fn* infection of macrophages is poorly understood, and whether *Fn* infection can induce the expression of IDO in macrophages and the effects of *Fn*-induced IDO on macrophage immune functions have not been investigated.

To elucidate the interactions between *Fn* and macrophages, we investigated the survival of both *Fn* and macrophages during *Fn* infection and identified a possible role for *Fn*-mediated IDO induction in limiting *Fn* multiplication inside macrophages and creating a microenvironment with suppressed lymphocyte immune responses to kill the infected host cells.

## Results

### *F*. *nucleatum* can invade and survive in THP-1-derived macrophages

**Fig. 1:**
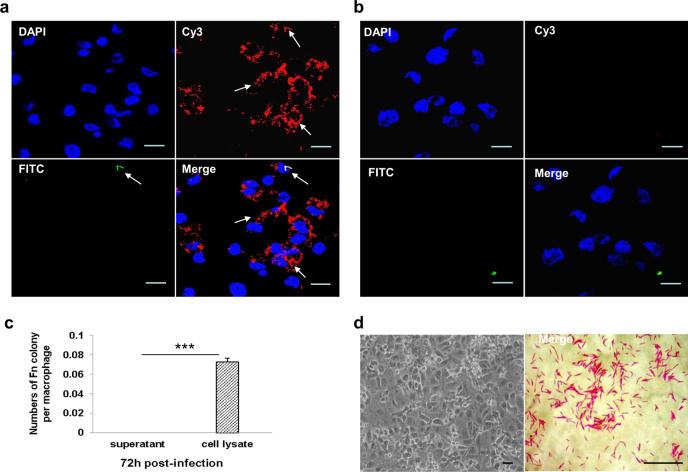
*F. nucleatum* invades THP-1-derived macrophages. THP-1-derived macrophages (dTHP1) were infected with *F. nucleatum* (*Fn*) at a MOI of 10:1 (bacteria:cells) for 48 h. Immunofluorescence staining of live *Fn* infection (**a**) and heat-killed *Fn* infection (**b**) were observed by confocal microscope (×60). **c** After 72 h co-culture, the recovery colonies numbers of average cell lysis and supernatant liquid. **d** Gram staining of *Fn* bacteria (×100) and *Fn*-infected dTHP1 cells (×20) were observed by light microscope. Bacteria external to the host cell were labeled with both Cy3 (red) and FITC (green), bacteria inside the cells were labeled with Cy3 (appearing red when channels were merged). Scale bar = 10 μm. ***P < 0.001

To investigate whether *Fn* can adhere to and invade macrophages, human THP-1-derived macrophages (dTHP1) were treated with live *Fn* bacteria at an multiplicity of infection (MOI) of 10:1 (bacteria:cells) and were incubated with the conventional cell culture method at 37 °C with 5% CO_2_. Bacteria invasion assays were carried out using an antibody-based differential staining method, all invasion experiments were performed under the aerobic condition. The specific immunofluorescence staining of *Fn* bacteria was confirmed by using mouse and human *Fn* polyclonal primary antibody respectively (Fig. S1). As shown in Fig. 1a, bacteria inside the cells were labeled with Cy3 (red), whereas *Fn* bacteria external to the host cell were labeled with both Cy3 and FITC (green, appearing yellow when channels were merged). Intracellular *Fn* were distributed mainly around the cell nucleus, and exhibited obvious morphological changes into short rod or spheres shapes in the cytoplasm of dTHP1 cells, whereas extracellular *Fn* showed normal fusiform rod shapes (Fig. 1a). In contrast, heat-killed *Fn* were not observed to enter host cells (Fig. 1b).

To further assay the survival of intracellular *Fn*, the *Fn*-infected dTHP1 were collected and lysed after 48 h post-infection, the culture supernatants and cell lysates were cultured in CDC blood agar anaerobically. Interestingly, large numbers of *Fn* bacterial colonies were observed from the cell lysates, whereas the culture supernatants of *Fn*-infected dTHP1 showed no bacterial growth (Fig. 1c). The Gram-negative bacteria which were isolated from the infected dTHP1 cells showed a fusiform morphology (Fig. 1d).

Those results indicated that *Fn* can invade and survival in the dTHP1 cell with the changed morphology. More importantly, those finding provided a convenient method for the co-culture of anaerobic intracellular bacteria and host cells under aerobic culture condition.

### *F*. *nucleatum* infection has little or no effect on the cell viability of THP-1-derived macrophages through activation of the PI3K/Akt and ERK signaling pathway

**Fig. 2:**
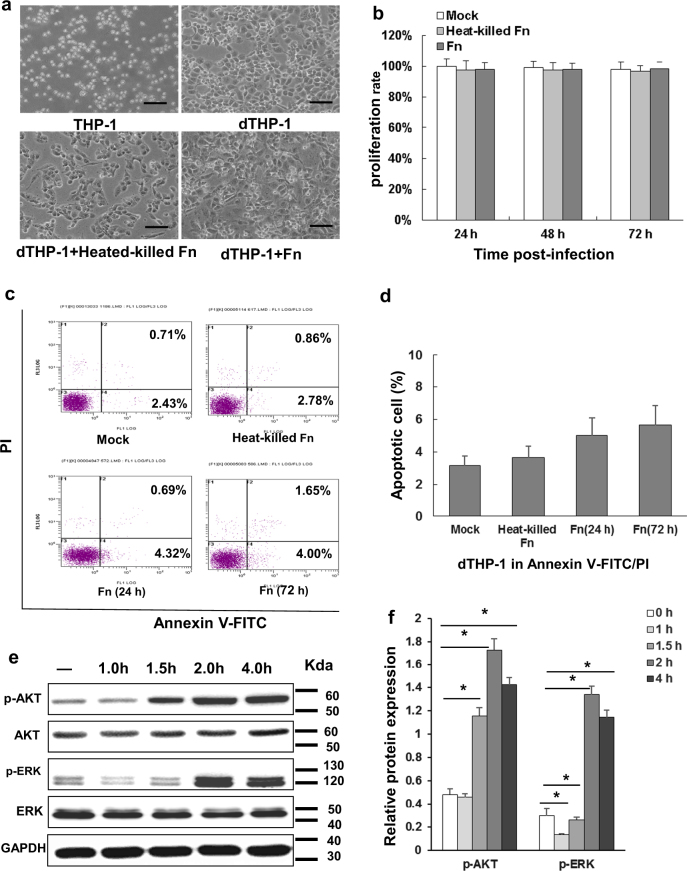
*F. nucleatum* infection exhibits little or no effect on the cell viability of THP-1-derived macrophages. dTHP1 cells were infected with dead *F. nucleatum* (*Fn*) (heat-killed-*Fn*, grey) or live *Fn* (*Fn*, dark grey) at a MOI of 10:1 (bacteria:cells) for the indicated time-points. **a** Morphology was observed at 72 h; **b** cell viability was measured by an MTT assay; **c**, **d** the apoptotic cells were analyzed by flow cytometry at 72 h; **e** the PI3K/AKT and ERK signaling pathway were analyzed by western blot from 1 h to 4 h and **f** quantitation was performed using pixel density analysis. Data indicate the mean ± standard deviation (SD) of triplicate-infected cultures. Bars represent the mean ± SD of the results from replicate measurements. *P < 0.05. Scale bar = 20 μm

To investigate whether *Fn* infection influences the survival of macrophages, dTHP1 cells were treated with *Fn* bacteria (MOI 10:1) and were incubated at 37 °C with 5% CO_2_. The dTHP1 cells exhibited obvious morphological changes into spindle shapes when infected with live or heat-killed *Fn* compared with the uninfected cells (Fig. 2a). However, MTT assays revealed that there was no significant difference in dTHP1 cell viability whether they were infected with either live or heat-killed *Fn* (Fig. 2b). In addition, in the presence of live or heat-killed *Fn* treated, dTHP1 cells exhibited no significant differences in the frequency of early apoptotic (FITC+PI−) or late apoptotic/necrotic (FITC+PI+) cells compared with uninfected cells according to flow cytometry (Fig. 2c, d). Furthermore, western blot assays revealed that live *Fn* infection induced a significant increase in Akt phosphorylation (p-AKT, ser473) and ERK phosphorylation (p-ERK, ERK1/2) after 1.5 h and 2 h of co-incubation, respectively. Maximal increases in the p-AKT and p-ERK levels occurred after 2–4 h (Fig. 2e, f). These results indicated that treated with live/heat-killed *Fn* exerted differentiation-like morphological changes, but not significant cytotoxicity on dTHP1 cells, and that macrophage apoptosis was inhibited during infection by activated PI3K/Akt and ERK signaling pathways.

### *F*. *nucleatum* infection induces classical activation of THP-1-derived macrophages, but has no effects on INF-γ expression

**Fig. 3:**
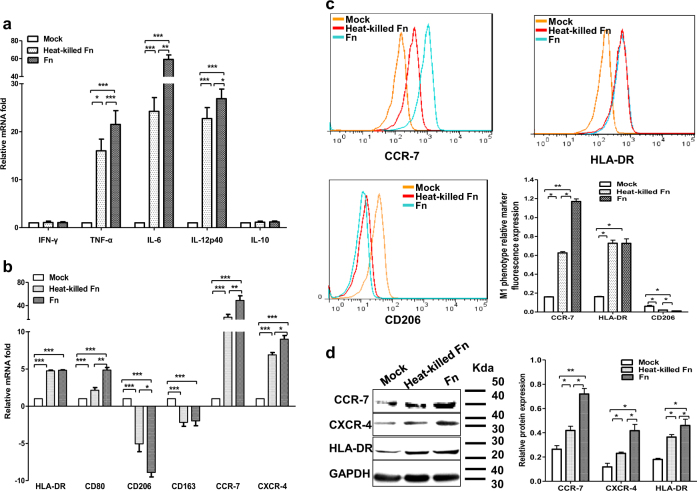
*F. nucleatum* infection induces classically activated THP-1-derived macrophages. The mRNA levels of **a** cytokines (INF-γ, TNF-α, IL-6, IL-12p40, IL-10); **b** MHC class II cell surface receptors (HLA-DR, CD80); M2-polarized phenotype markers (CD206, CD163); and chemokine receptors (CCR7, CXCR4) were assessed by qRT-PCR in dTHP1 cells infected with heat-killed-*Fn* or live *Fn* at an MOI of 10:1 for 24 h. The expression of the M1-polarized phenotype marker CCR7, CXCR4, and HLA-DR in dTHP1 cells with live/heated-killed *Fn* at an MOI of 10:1 for 48 h was analyzed by flow cytometry (**c**) and western blot (**d**). Bars represent the mean ± SD of the results from replicate measurements. *P < 0.05, **P < 0.01, ***P < 0.001

To evaluate whether live *Fn* infection leads to the induction of activated or polarized dTHP1 cells, an array of five different cytokines, two chemokine receptors, and two MHC class II cell surface receptors, which are typically produced during bacterial infections, was established, and mRNA expression was analyzed by qRT-PCR. The relative fold change of mRNA was calculated relative to the uninfected dTHP1 cells. The pro-inflammatory cytokines IL-6, IL-12p40, and TNFα (Fig. 3a); the chemokine receptors CCR7 and CXCR4 (Fig. 3b); and the MHC class II cell surface receptors HLA-DR and CD80 (Fig. 3b) were highly induced by live/heat-killed *Fn*, whereas the expression of INF-γ and anti-inflammatory cytokine IL-10 remained unaffected (Fig. 3a). Moreover, macrophage mannose receptor CD206 and scavenger receptor CD163, both of which are M2-polarized phenotype markers, exhibited reduced expression in *Fn*-dTHP1 cells (Fig. 3b). In addition, western blot and flow cytometry assays revealed that the expression of the macrophage M1 phenotype markers CCR7, CXCR4, and HLA-DR were increased with live *Fn* or heated-kill *Fn* treated (Fig. 3c, d). These results indicated that infection with *Fn* induces classically activated (M1-polarized) macrophages with the exception of INF-γ expression.

### *F*. *nucleatum* infection induces IDO expression in THP-1-derived macrophages

**Fig. 4:**
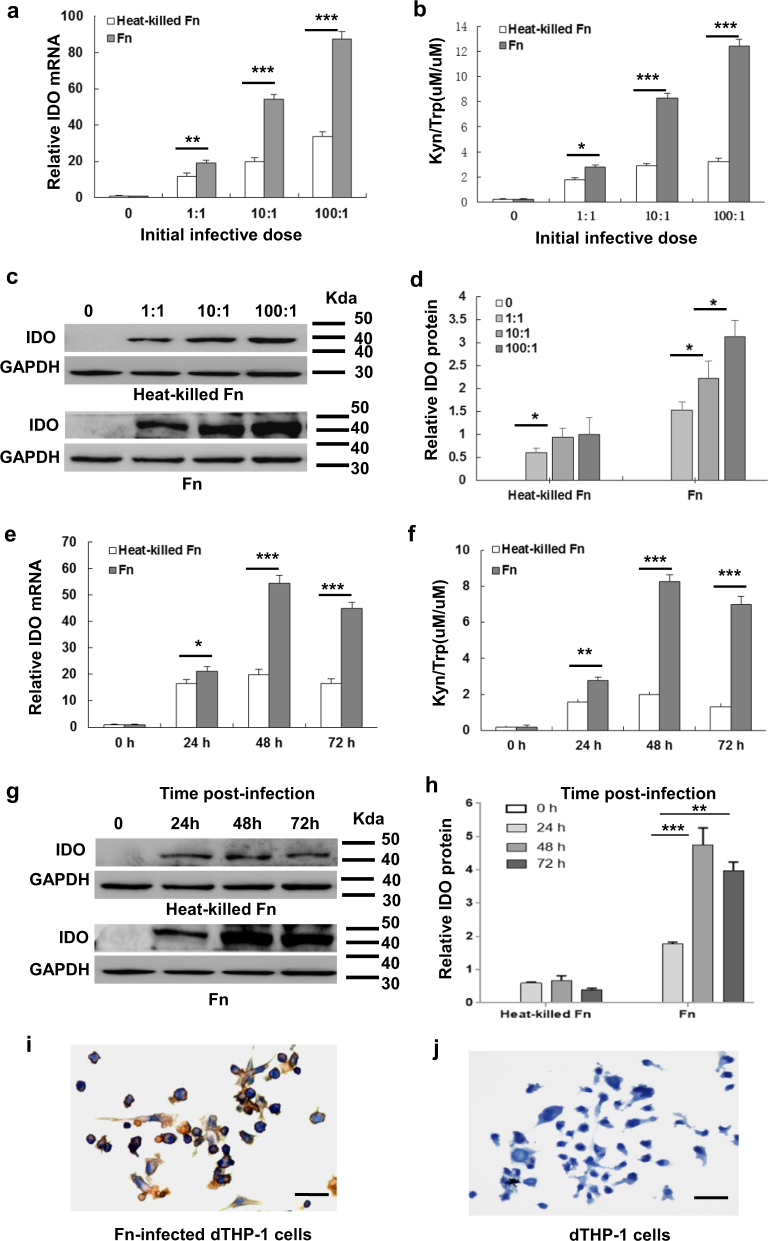
*F. nucleatum* infection induces IDO expression in THP-1-derived macrophages in a dose-dependent and time-dependent manner. **a** IDO mRNA expression assessed by qRT-PCR, **b** induction of IDO enzymatic activity by HPLC, and **c**, **d** representative western blots for IDO protein expression in dTHP1 cells infected with live *Fn* or heat-killed-*Fn* for 48 h at different dosages; **e** IDO mRNA expression assessed by qRT-PCR, **f** induction of IDO enzymatic activity by HPLC and **g**, **h** representative western blots for IDO protein expression in dTHP1 cells infected with live *Fn* or heat-killed-*Fn* at a MOI of 10:1 for the indicated time-points; **i** *Fn*-infected dTHP1 cells exhibit positive cytoplasmic staining for IDO at a MOI of 10:1 for 48 h and **j** negative staining for uninfected-dTHP1 cells as detected by immunohistochemistry. Bars represent the mean ± SD of the results from replicate measurements. ^Below the detection limit. *P < 0.05, **P < 0.01, ***P < 0.001. Scale bar = 20 μm

IDO expression in dTHP1 cells treated with live or heat-killed *Fn* was assessed by qRT-PCR and western blot analysis, respectively. The IDO mRNA and protein assays revealed that IDO can be induced in dTHP1 cells by treatment with live/heat-killed *Fn*. Moreover, live *Fn* infection exhibited higher expression of IDO than treatment with heat-killed *Fn* (Fig. 4a–h). Infection with low-dose live or heat-killed *Fn* at an MOI of 1:1 for 24 h was able to induce IDO expression. IDO was further increased in a time-dependent manner, peaking at 48 h (Fig. 4g, h), and a dose-dependent manner, peaking at an MOI of 100:1 (Fig. 4c, d). The enzymatic activity of IDO was also investigated by HPLC. The enzymatic activity of IDO was almost undetectable in the supernatant of dTHP1 cells, but was observed in cells infected with live or heat-killed *Fn* (Fig. 4b, f). These enzymatic activity results are consistent with the qPCR and western blotting results. Figure 4i–j demonstrates that some *Fn*-dTHP1 cells exhibited positive cytoplasmic IDO staining by immunohistochemical analysis. Those results confirmed that live or heat-killed *Fn* treated can induce IDO expression in human macrophages and that live *Fn* infection induced a higher level of IDO.

### Involvement of IL-6 and TNF-α in the induction of IDO by ***F****. nucleatum* infection of THP-1-derived macrophages

**Fig. 5:**
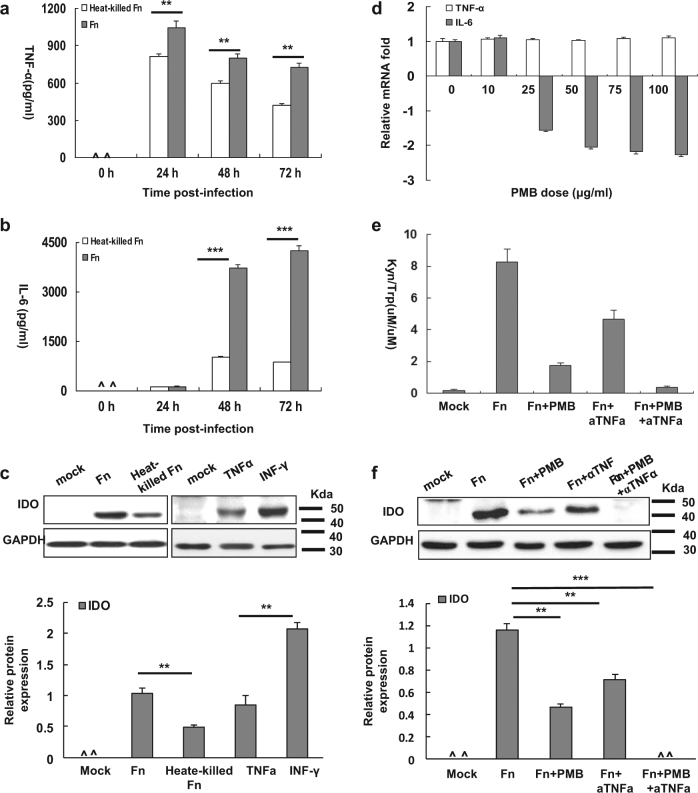
Involvement of IL-6 and TNF-α in the induction of IDO by *F. nucleatum*-infected THP-1 cells. TNF-α (**a**) and IL-6 (**b**) levels in the supernatants of *Fn*- or heat-killed *Fn*-infected dTHP1 cells at an MOI of 10:1 for the indicated times. **c** IDO expression was assessed by Western blot at 48 h. **d** mRNA expression of IL-6 and TNF-α level in the presence of PMB with the indicated dosages. IDO enzymatic activity in supernatants (**e**) and representative western blots for IDO in cells lysates (**f**) of live *Fn*-infected dTHP1 cells (MOI: 10:1) in the presence of neutralizing antibodies to TNF-α and/or PBM for 48 h. Bars represent the mean ± SD of the results from triplicate determinations. ^Below the detection limit. *P < 0.05, **P < 0.01, *** *P* < 0.001

To investigate the cytokines that are involved in the induction of IDO in *Fn*-infected dTHP1 cells (*Fn*-dTHP1), cytokines were measured using a cytokine bead array. As shown in Table S1, the INF-γ, IL-2, IL-4, and IL-10 concentrations were below the limits of detection until 72 h in the supernatant of dTHP1 cells infected with live or heat-killed *Fn*. High levels of IL-6 and TNF-α were detected in the supernatants of both live *Fn*-dTHP1 and heat-killed *Fn*-dTHP1 cells. Moreover, IL-6 and TNF-α were significantly increased in the supernatant of live *Fn*-dTHP1 cells relative to the supernatant of heat-killed *Fn*-dTHP1 cells (*P* < 0.001) (Fig. 5a, b). These findings are consistent with the IDO expression levels measured in treated live or heated-killed *Fn* by western blot assay at 48 h (Fig. 5c).

In addition, TNF-α (1043.78 ± 53.23 pg/ml) levels reached their peak at 24 h post-live *Fn* infection and substantially decreased. However, IL-6 was substantially elevated and reached its peak at 72 h post-live *Fn* infection (4256.35 ± 135.63 pg/ml) (Fig. 5a, b). Given that lipopolysaccharide (LPS) induces the production of TNF-α and IL-6, which are potent inducers of IDO expression, we examined the possible involvement of LPS or these two cytokines in IDO expression induced by live *Fn* infection using neutralizing assays. LPS neutralized by polymyxin B (PMB, >25 μg/ml) decreased the IL-6 mRNA level, but had no effect on the TNF-α level, even at the highest dose of PMB (100 μg/ml) (Fig. 5d). Higher levels of PMB (>100 μg/ml) were cytotoxic to dTHP1 cells (date not show).

Furthermore, LPS neutralized by the highest dose of PMB (100 μg/ml) markedly reduced the expression of IDO and enzyme activity, but was unable to completely inhibit IDO expression (Fig. 5e, f). Moreover, neutralizing antibodies against TNF-α significantly blocked the increased IDO expression and enzyme activity induced by live *Fn* infection, and the combination of PMB and TNF-α antibodies almost completely blocked the induction of IDO protein and its enzyme activity (Fig. 5e, f). These data indicate that *Fn* infection is able to induce several pro-inflammatory cytokines by LPS in dTHP1 cells with the exception of IFN-γ, and LPS, and that the expression TNF-α stimulated by *Fn* infection was the most involved cytokine in the induction of IDO.

### *F*. *nucleatum* undergoes limited proliferation in the low-tryptophan environment of IDO-induced THP-1-derived macrophages

**Fig. 6:**
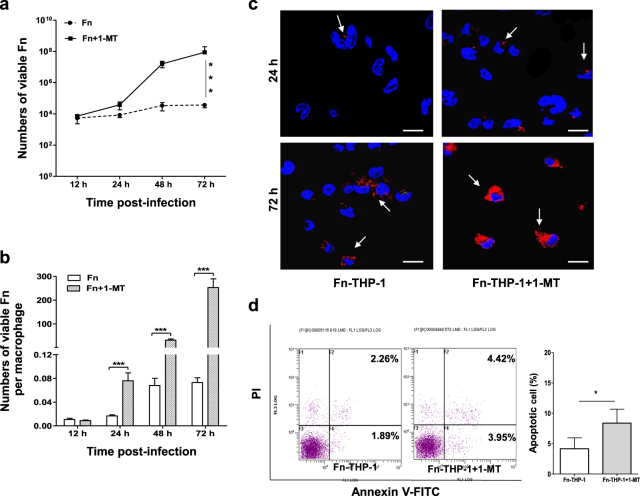
*F. nucleatum* survives and undergoes limited intracellular proliferation in THP-1-derived macrophages. Intracellular bacteria proliferation was assessed by gentamycin protection assay. *Fn*-dTHP1 cells with or without treated by 1-MT were lysed at the indicated time-points after infection, and the numbers of total viable bacteria (**a**) and viable bacteria per macrophage (**b**) were determined by the serial dilution method. **c** Immunofluorescence staining of intracellular *Fn* (red) was observed by confocal microscope at 24 h and 72 h (×60). **d** The apoptotic cells were analyzed by flow cytometry at 72 h. Bars represent the mean ± SD of the results from triplicate determinations. *P < 0.05, **P < 0.01, ***P < 0.001. Scale bar = 20 μm

To assess the ability of *Fn* to multiply inside macrophages, a gentamycin protection assay was performed. *Fn* was able to survive up to 72 h in dTHP1 cells, although the mean surviving intracellular bacteria were depleted to a small fraction of the inoculums (0.073 living intracellular bacteria per macrophage after 72 h post-infection of 10 originally inoculated bacterial cells per macrophage, Fig. 6a). Whereas, strictly extracellular anaerobic bacteria would die if they were no invasion. Most interestingly, the IDO-specific inhibitor 1-methyl-l-tryptophan (1-MT) relieved the inhibition of *Fn* proliferation inside macrophages. With the treatment of 1-MT (100 µM), *Fn* grew quickly from 24 to 48 h post-infection, resulting in up to >100 living intracellular bacteria per macrophage after 72 h post-infection (Fig. 6a, b). Immunofluorescence staining showed the marked increase of intracellular *Fn* (red) after 72 h post-infection with the treatment of 1-MT (Fig. 6c). In addition, *Fn*-dTHP1 cells treated with 1-MT exhibited only a slight elevation in the rates of apoptosis and necrosis after 72 h post-infection according to flow cytometry (Fig. 6d).

**Fig. 7:**
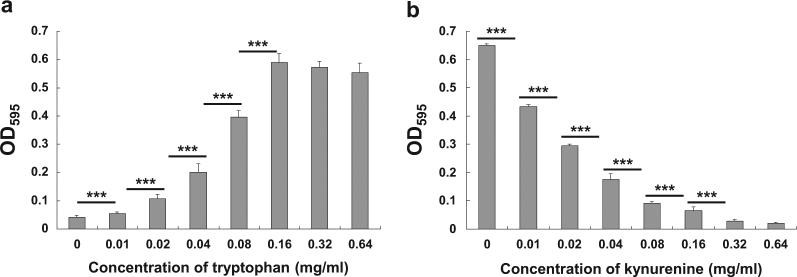
Growth of *F. nucleatum* is regulated by tryptophan and kynurenine in a dose-dependent manner. **a** *Fn* grew in a tryptophan dose-dependent manner. **b** The growth of *Fn* was inhibited with kynurenine in a dose-dependent manner. Bacterial growth was assessed in enriched BHI broth by spectrophotometry. Columns indicate the mean of six replicate measurements, and bars indicate the SD (**P < 0.01, ***P < 0.001)

Given that IDO generates a tryptophan-deficient environment to inhibit *Fn* proliferation, we cultured *Fn* in tryptophan-enriched or kynurenine-enriched BHI medium in vitro. Tryptophan promotes the growth of *Fn* in a dose-dependent manner; the minimum effective tryptophan concentration is 0.02 mg/ml, and the effect peaks at 0.32 mg/ml (Fig. 7a). The growing *Fn* population doubles with enriched tryptophan from 0.02 to 0.18 mg/ml, and an about 1.5-fold increase is observed from 0.18 to 0.36 mg/ml. Furthermore, kynurenine has the opposite effect and inhibits the growth of *Fn* in a dose-dependent manner (Fig. 7b). *Fn* growth decreased by about 1.5-fold at the low Kyn concentration from 0.01 to 0.04 mg/ml, decreased about twofold at the high concentration from 0.04 to 0.16 mg/ml and was almost completely inhibited at the dose of 0.36 mg/ml.

Those results indicate that *Fn* is able to survive and undergo limited intracellular proliferation in macrophages. The growth of *Fn* inside macrophages indicated that the intracellular proliferation of *Fn* was limited due to the concentration of tryptophan and kynurenine induced by the expression of IDO. Moreover, blocking IDO enzyme activity markedly promoted intracellular proliferation of *Fn*.

### *F*. *nucleatum*-infected dTHP1 cells escape killing by impairing the cytolytic function of peripheral blood lymphocytes

**Fig. 8:**
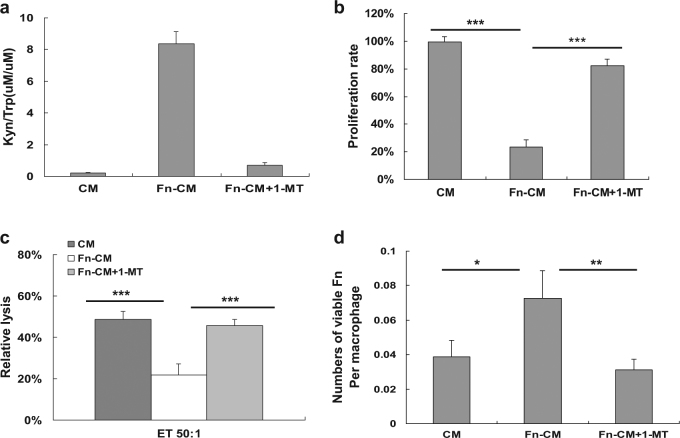
Effects of IDO in the conditional media (CM) of Fn-dTHP1 cells on the proliferation and cytolytic activity of lymphocytes cells. **a** Concentrations of tryptophan (Kyn) and kynurenine (Try)were measured by HPLC in the supernatants of dTHP1 or *Fn*-dTHP1 cells (MOI 10:1), supernatants of dTHP1 cells (CM), supernatants of *Fn*-infected cells (CM-Fn), or infected cell supernatants plus 1-MT (100 μM) (*Fn*-CM + 1-MT) for 48 h. **b** MTT proliferation assay of Jurkat T cells with indicated CM at 48 h. **c** PBLs derived from PBMCs were activated by IL-2 and cultured in the CM, CM-*Fn*, or the CM with 1-MT (100 μM) (*Fn*-CM + 1-MT) for 48 h. Cytolytic activity against the target cell:*Fn*-dTHP1 was evaluated using a standard LDH release assay. **d** Live *Fn* numbers were determined by a gentamycin protection assay in a mixture of *Fn*-dTHP1 and PBLs. The E:T ratios are 50:1. Bars represent the mean ± SD of the results from three or six independent experiments. *P < 0.05, **P < 0.01, ***P < 0.001

To investigate whether IDO produced by *Fn*-dTHP1 has an effect on the proliferation activity of T lymphocyte cells, an MTT assay was conducted using the supernatant of dTHP1 cells as conditioned medium (CM). CM from live *Fn*-dTHP1 (*Fn*-CM) exhibited IDO enzyme activity, whereas CM from uninfected dTHP1 exhibited almost undetectable IDO enzyme activity (Fig. 8a). In addition, the functional IDO enzyme activity in 1-MT-treated CM from *Fn*-dTHP1 cells was markedly inhibited by 1-MT. Moreover, CM from *Fn*-dTHP1 cells markedly inhibited the proliferation of human Jurkat T lymphocyte cells (*P* < 0.001), and the proliferation was almost completely restored (82.8%) by CM from *Fn*-dTHP1 cells with the addition of 1-MT (Fig. 8b).

To investigate the anti-*Fn* effect of cytotoxicity against infected macrophages by peripheral blood leukocyte (PBL), an LDH release assay was conducted using dTHP1 cells infected by *Fn* for 48 h as targets. PBLs from three healthy volunteers whose feces exhibited high levels of *Fn*-DNA by PCR detection were stimulated by IL-2 for 96 h as effectors. PBLs lysed the target cells at 50:1 E/T ratios when exposed to CM, whereas the lysis rate was remarkably reduced when the PBLs cells were exposed to CM from *Fn*-dTHP1 cells (*P* < 0.001). The cytolytic activity of PBLs cells was effectively restored when exposed to CM from *Fn*-dTHP1 cells in the presence of 1-MT (Fig. 8c). Next, the mixture of targets and effectors cells were collected and lysed to release intracellular bacteria. The CFU counts of Fn (0.072 per macrophage) were increased when PBLs cells were exposed to CM from Fn-dTHP1 cells, whereas the CFU counts of *Fn* (0.031 per macrophage) were effectively restored when exposed to CM from *Fn*-dTHP1 cells in the presence of 1-MT (Fig. 8d).

These results indicated that exposure to the microenvironment created by *Fn*-infected macrophages severely reduced lymphocyte cell survival and impaired the cytotoxic function, providing a potential mechanism for the immune evasion and spread of *Fn* bacteria via infected macrophages.

## Discussion

In this study, we identified *Fn* as an obligate anaerobe that can invade and multiply inside host cells under aerobic co-culture conditions. *Fn* has been reported previously to survive during the invasion of oxygenated tissues in host oral cavities by adapting to oxidative stress with enhanced pathogenicity^19^. Generally, intracellular oxygen restriction can impair the mitochondrial respiratory chain to inhibit aerobic bactericidal activity inside macrophages, such as in the case of *L. monocytogenes* or *M. tuberculosis* infection^20^, but this response may provide a suitable hypoxic microenvironment for anaerobic intracellular bacteria. In our study, intracellular *Fn* exhibited morphological changes into the short rod or spheres shapes that may be responsive to intracellular environmental stress. Similarly, intracellular *Helicobacter pylori* have been observed the morphology transition from normal helical bacillary to a coccoid shape^21^. What’s more, *Fn* exhibits limited proliferation when it is co-cultured with host cells under aerobic conditions, which demonstrates that the intracellular environment is more suitable than the extracellular environment for the survival of *Fn* in an un-strict anaerobic environment. This finding provides a convenient research methodology for the interaction of anaerobic pathogens with host cells.

Microbial infections often elicit programmed cell death as part of the host defense system or as a part of the survival strategy of the pathogen, but some intracellular bacteria manipulate the host death and survival pathways to enhance their replication and survival by blocking or inhibiting the macrophage apoptotic responses of the mitochondrial pro-death, NF-κB–dependent pro-survival, and inflammasome-dependent host cell death pathways during infection^22^. In our study, *Fn* infection kept host cells alive up to 72 h, wheraes they reproduced by suppressing host cells apoptosis via the PI3K pathway, suggesting macrophage-mediated dissemination of *Fn* infection in the body.

Canonical Th1 cytokine IFN-γ is critical for innate and adaptive immunity, especially in response to intracellular bacterial infections^23^. Macrophages activated by IFN-γ exhibit increased pinocytosis and receptor-mediated phagocytosis as well as an enhanced microbial killing ability to restrain intracellular bacterial replication. *Fn*-infected macrophages were activated in a classically M1-polarized manner in our study, but with the absence IFN-γ secretion, which alleviated the effect of host cells against intracellular bacteria. Similar results were observed in *Fn*-infected natural killer (NK) cells with inhibition of IFN-γ secretion; infection inhibited NK cell cytotoxicity via the interaction of the Fap2 protein of *Fn* with TIGIT ^24^.

Similarly to other intracellular bacteria*, Fn* infection induced high TNF-α and IL-6 secretion. A continuous increase of IL-6 secretion during infection mediates host defense and cell survival in different bacterial infections^25^. Furthermore, TNF-α is produced early in bacterial infection as a Trojan horse to ensure their intracellular replication^26^. A wide spectrum of microbes has acquired elegant mechanisms to overcome or deflect the host responses mediated by TNF-α^27^. TNF-α-mediated activation of IDO has been reported in microbial infections^28^. Our data indicate that *Fn* induces IDO expression in response to TNF-α and LPS stimuli in dTHP1 cells. These results are consistent with previous studies that infection with viruses, including HIV and EBV, as well as with some bacteria, such as *Haemophilus ducreyi* and *L. monocytogenes*, induced IDO expression in macrophages^29–32^. In addition, TNF-α altered type 1 immune activation in part by suppressing T-cell proliferation during mycobacterial infection ^26^.

IDO activation in APCs can potently inhibit the immune response by which tryptophan is depleted in cells in response to the infection, which may reflect an antiparasitic mechanism in humans^26^. As a tumor-associated bacterium, *Fn* exhibits similar nutritional regulation with respect to tryptophan metabolism, and both *Fn* and tumors are sensitive to tryptophan-depleted microenvironments. It is interesting that kynurenine exhibits an inhibitory effect on the growth of *Fn*, which is also observed in *L. monocytogenes*^33^. The IDO-induced high kynurenine and low-tryptophan microenvironment limited the proliferation of Fn and impaired the function of T-cells, which might downregulate anti-*Fn* or anti-CTL cell responses, leading to *Fn* persistence in vivo.

Immune checkpoint blockade therapy has opened a new therapeutic era for cancer therapy by activating anti-tumor T-cell immunity. IDO inhibitors such as D-1-MT, which can suppress the growth of tumor cells by relieving T-cell suppression, have been applied in clinical trials in some tumors^34^. Given that IDO blockade relieved intracellular bacterial and T-cell suppression, we asked whether IDO inhibitors affected *Fn* multiplication. Indeed, we found that *Fn*-infected dTHP1 cells exhibited a significantly greater bacterial load after treatment with 1-MT, demonstrating that repression of IDO relieved the growth of bacteria, largely inside macrophages. Similarly, some anti-inflammatory drug, such as TNFα blockers, which have been used for the treatment of certain patients with rheumatoid arthritis and Crohn’s disease, contain warnings for serious infections from some associated disease-causing bacterial pathogens, including *Salmonella, Legionella*, and *Listeria*^35,36^. These data suggest that therapy with IDO inhibitors might result in the exacerbation of infection and bacteria spread throughout the body via infected macrophages in some *Fn*-associated cancers, such as CRC.

Taken together, these findings indicate that *Fn* is able to survival and undergo limited intracellular proliferation in macrophages, and the induction of IDO expression creates a tryptophan-deficient and kynurenine-rich toxic microenvironment inside macrophages to inhibit the proliferation of both intracellular *Fn* bacteria and T lymphocytes in the microenvironment, thereby allowing *Fn*-infected macrophages to escape attack by CTL cells. Moreover, *Fn* inside infected macrophages evade cell-intrinsic death by activating the PI3K and ERK pathways to inhibit host cell apoptosis. In conclusion, *Fn* escape is regulated by nutritional requirements that are similar to those of T cells. Our findings suggest that IDO inhibitors may aggravate infection in *Fn*-associated tumor therapy.

## Materials and methods

### Bacterial culture

*F. nucleatum* (*Fn*) strain ATCC 25586 was purchased from the China General Microbiological Culture Collection Center (CGMCC, Beijing, China). The organisms were grown anaerobically (AnaeroPack, Bio-Merieux, France) at 37 °C for 72 h on CDC anaerobic blood agar plates (Guangzhou detgerm Microbiology Technology Co. Ltd, Guangzhou, China) or 48 h in brain heart infusion (BHI, Oxoid, Hampshire, UK) broth medium before harvesting. Heat-killed (dead) *Fn* was made by heating at 100 °C for 10 min. Then, live/heat-killed *Fn* were centrifuged and suspended to 1 × 10^8^ colony-forming units (CFUs)/ml with RPMI 1640 (Hyclone Labs, Logan, UT) for infection experiments

The influence of tryptophan (Trp, Sigma-Aldrich, St. Louis, MO) and kynurenine (Kyn, Sigma-Aldrich) on the growth of Fn was assayed using a method described as follows: *Fn* were harvested in the exponential growth phase and subsequently resuspended and diluted with BHI medium to the appropriate concentration. The twofold serially diluted samples with Try or Kyn (0.01–0.64 mg/ml) were placed into a flat-bottom 96-well microtiter plate and incubated anaerobically for 48 h at 37 °C. After incubation, the bacterial cell culture was measured by spectrophotometry with an iMark microplate absorbance reader (Bio-Rad, Philadelphia, USA) at 595 nm to assess bacterial growth.

### Cell culture and treated

Peripheral blood mononuclear cells (PBMCs) were isolated by Ficoll-Paque plus gradient centrifugation of leukopacks derived from three healthy volunteers with positive Fn-DNA in stool. Informed consent was obtained from the volunteers prior to participation in accordance with the human experimentation guidelines of the Institute Research Ethics Committee of the Cancer Centre, Sun Yat-Sen University (No: GZR2012-123). Monocytes and phagocytes were removed by adherence to plastic by culturing PBMCs in RPMI 1640 containing 50 U/ml IL-2 (Peprotech, Rocky Hill, USA) for 4–5 h. The non-adherent peripheral blood lymphocyte (PBL) fraction was harvested and cultured with 100 U/ml IL-2 in complete RPMI 1640 medium (containing 10% FCS, 100 U/ml penicillin and 100 μg/ml streptomycin).

The human monocyte cell line THP-1 and T lymphocyte cell line Jurkat (Cell Bank of Chinese Academy of Sciences, Shanghai, China) were grown in RPMI 1640 supplemented with 10% fetal bovine serum (FBS). THP-1 monocytes were differentiated into macrophages (THP-1 derived macrophages, dTHP1) by treatment with 10 nM PMA (phorbol 12-myristate 13-acetate, Sigma-Aldrich) for 48 h.

For bacterial and host cells co-culture experiments, dTHP1 cells were added at the indicated concentrations to live/heat-killed *Fn* and cultured for the indicated times under a humidified 5 % CO_2_ atmosphere at 37 °C in a CO_2_ incubator. For blocking experiments, dTHP1 cells were pre-incubated with 10 µg/ml of neutralizing antibodies against TNF-α mouse IgG1 [mIgG1] (eBioscience, San Diego, CA) or 100 µg/ml of polymyxin B (PMB, Sigma-Aldrich) for 2 h at 37 °C before treated.

### CM treatment

dTHP1 cells were cultured in 6-well plates (1.5 × 10^6^ cells per well) in the absence or presence of live/heat-killed *Fn* for 24 h, and the medium was then replaced by fresh medium, with or without 100 µM 1-methyl-d-tryptophan (1-MT, Sigma-Aldrich). Twenty-four hours after medium replacement, the culture medium was harvested as CM and used for the incubation of Jurkat T cells or PBLs.

### Cell viability assay

Cell proliferation or viability was measured using an MTT cell proliferation kit (Beyotime Biotechnology, Shanghai, China) following the manufacturer’s instructions. Briefly, cells were seeded into 96-well plates at a density of 5 × 10^4^ cells per well and were cultured for 12 h. Then, cells were infected with live/heat-killed bacteria or treated with CM for the indicated times. The absorbance was measured at 570 nm by an iMark Microplate Absorbance Reader.

### Quantitative RT-PCR

The total mRNA of the cells was extracted after treatment for the indicated time. First strand cDNA synthesis was carried out from 800 ng of total RNA. The quantification of the target and reference (18s RNA) genes was performed in triplicate using a LightCycler® 480 II (Roche Diagnostics, Mannheim, Germany). The primers used in the real-time PCR reaction are presented in Table S2.

### Immunoblotting

Total protein extracts were extracted by using a lysis buffer and protease inhibitor (Beyotime Biotechnology) from cultured cells after treatment for the indicated time intervals. Equivalent protein amounts were separated by SDS-PAGE and transferred onto polyvinylidene difluoride membranes. After being blocked with 5% non-fat dry milk in PBS containing 0.05% Tween-20, the blotted membranes were incubated with polyclonal antibodies against CCR7, CXCR4 (1:800, Bioworld, St. Louis, MN), HLA-DR(1:800, Abcam, Cambridge, UK) or monoclonal antibodies against IDO (1:1000, Cell Signaling Technology Inc., Beverly, MA), AKT (1:1000, Abcam), p-AKT (1:1000, Cell Signaling Technology Inc.), ERK1/2 (1:1000, Cell Signaling Technology Inc.), or p-ERK1/2 (1:1000, Cell Signaling Technology Inc.), as well as a horseradish peroxidase-conjugated secondary antibody. The GAPDH protein levels were also determined using a specific antibody (1:5000, Bioworld) as a loading control. Western blot signal was quantified by ImageJ software.

### Measurement of IDO activity

The cell culture medium was mixed with trichloroacetic acid and then centrifuged. Subsequently, the supernatant was injected onto a C-18 column and eluted with KH_2_PO_4_. The concentrations of Trp and Kyn were analyzed by HPLC (Waters). Trp was measured by the detection of its native fluorescence at 285-nm excitation and 365-nm emission wavelength. Kyn was detected by UV absorption at the 360-nm wavelength in the same chromatographic run, and the results were processed using Breeze version 3.30 SPA software. IDO activity was determined by calculating the Kyn to Try ratio (Kyn/Trp, μM/μM).

### Immunofluorescence

Bacteria invasion assays were carried out using a differential staining immunofluorescence procedure as previously similar described^34^. Briefly, *Fn*-infected cells were washed with PBS at least three times to remove non-adherent bacteria and then fixed with 4% paraformaldehyde, and blocked in 10% (v/v) normal goat serum. Human anti-*Fn* polyclonal antibody (home-made, purified IgG from serum of a CRC patient with high anti-*Fn* level^35^, 1:100) was incubated overnight at 4 °C. Then, cells were permeabilized by the addition of 0.5% Triton X-100, and incubated with prepared mouse anti-*Fn* polyclonal antibodies (home-made, mouse immunized by heat-killed *Fn*, 1:100). Following this, cells were labeled with Cy3-labeled goat anti-mouse IgG (1:400, Boster, China) as well as FITC-labeled goat anti-human (1:50, Boster, China) for 30 min at 37 °C. DAPI staining for nuclear standing <10 min. After every step, cells were washed three times with PBS. Finally, cells were imaged at ×60 magnification using the Zeiss LSM710 confocal microscope (Zeiss, Oberkochen, Germany). Using this protocol, bacteria external to the host cell were labeled with both Cy3 and FITC, whereas bacteria inside the cells were labeled with Cy3 only (appearing only red when channels were merged).

### Immunohistochemistry

The sections were immersed in a 3% hydrogen peroxide solution for 10 min to block endogenous peroxidase activity and were incubated with the primary antibody rabbit anti-human IDO (1:700, Cell Signaling Technology, Inc.) at 4 °C overnight. A negative control was performed by replacing the primary antibody with PBS. The sections were then incubated with a horseradish peroxidase-labeled secondary antibody (1:100, Boster, Wuhan, China) at room temperature for 120 min. Finally, the signal was developed for visualization with 3,3′-diaminobenzidine tetrahydrochloride, and all of the slides were counterstained with hematoxylin.

### Flow cytometry

dTHP1 cells that were 80% confluent were treated with *Fn* for 24 h. Cells were observed under an inverted microscope (Nikon TE 300), harvested in 5 mM EDTA in PBS and washed. For analysis of CCR7, CD206, and HLA-DR, cells were resuspended with 300 µl of PBS and 5 µl of PE-conjugated anti-human CCR7 and CD206 (eBioscience) or FITC-conjugated anti-human leukocyte antigen (HLA)-DR (BD Biosciences, Erembodegem, Belgium) for 15 min and then analyzed by a FACS Calibur (Beckman-Coulter, Miami, USA). For analysis of cell apoptosis, cells were resuspended with annexin-binding buffer and then stained with annexin V and propidium iodide (PI) according to the manufacturer’s instructions (BD Biosciences). Apoptotic cells were analyzed by a FACS Calibur (Beckman-Coulter).

### Cytokine analysis

The qualification of cytokines in the CM was performed using a BD™ Cytometric Bead Array panel kit (BD Biosciences). The analytes included in the 6-plex kit were as follows: IL-2, IL-4, IL-6, IL-10, gamma interferon (IFN-γ), and TNF-α. The 6 cytokines were measured by flow cytometry according to the manufacturer’s instructions.

### Intracellular survival assays

Bacterial infection for intracellular entry and proliferation was assessed according to the gentamycin protection assay. Briefly, dTHP1 cells were seeded in a 24-well plate at a density of 1 × 10^6^ cells per well in RPMI 1640 with 10% FBS. After 24 h, cells were infected for 2 h with live *Fn* bacteria at a MOI of 10:1. For blocking experiments, dTHP1 cells were pre-incubated with 1-MT (100 μM) for 2 h prior to infection. After infection, dTHP1 cells were washed three times with RPMI 1640 and then incubated for 2 h in RPMI 1640 containing 10% FBS and gentamicin (50 μg/ml) to remove extracellular *Fn*. Subsequently, the culture wells were transferred to RPMI medium containing a lower dose of gentamycin (10 μg/ml) and 0 or 10 µM 1-MT and cultured for the indicated times. Ultimately, dTHP1 cells were lysed with 0.1% Triton X-100 and 0.01% SDS in PBS, and serial dilutions were spread onto anaerobic blood agar plates to quantify the number of internalized (intracellular) bacteria with a mean of six replicate measurements.

### Cytotoxicity assay

The cytotoxic activity of the PBLs was determined by a standard lactate dehydrogenase (LDH) release assay using a CytoTox 96® kit (Beyotime Biotechnology, China) following the manufacturer’s instructions. Briefly, the *Fn*-infected dTHP1 cells (5 × 10^4^ cells/well) were cultured as target cells. IL-2-stimulated PBLs from three healthy volunteers were incubated in different CM as the treated effector cells. The target cell and effector cell suspensions were co-cultured at effector-to-target (E:T) ratios of 50:1. After 4 h of incubation, the release of LDH into the supernatant was quantified by recording the absorbance at 490 nm. The percentage of cytotoxicity was calculated as described by the manufacturer.

### Statistical analysis

All values are presented as the mean ± SD. Paired *t* tests were used to analyse data unless otherwise indicated. We report the nominal *P* value for each comparison without adjusting for multiple testing. A *P* value < 0.05 was considered statistically significant.

## Electronic supplementary material

Supporting information
