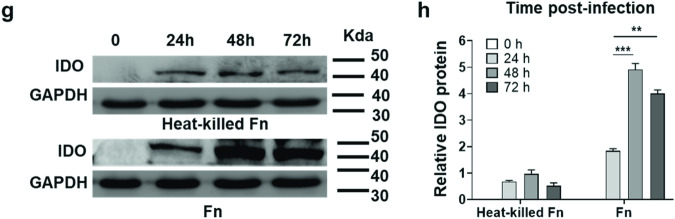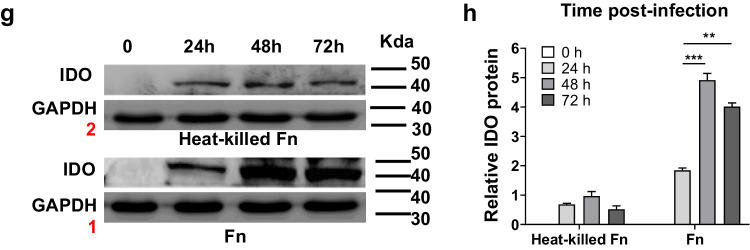# Correction to: Indoleamine 2,3-dioxygenase expression regulates the survival and proliferation of *Fusobacterium nucleatum* in THP-1-derived macrophages

**DOI:** 10.1038/s41419-024-06516-w

**Published:** 2024-02-12

**Authors:** Ying Xue, Han Xiao, Songhe Guo, Banglao Xu, Yuehua Liao, Yixian Wu, Ge Zhang

**Affiliations:** 1 School of Public Health (Shenzhen), Sun Yat-sen University, Guangdong, China; 2 Department of Microbial and Biochemical Pharmacy, School of Pharmaceutical Sciences, Sun Yat-sen University, Guangzhou, China; 3 Department of Clinical Laboratory Medicine, Guangzhou First Municipal People’s Hospital, Guangzhou Medical University, Guangzhou, China

Correction to: *Cell Death and Disease* 10.1038/s41419-018-0389-0, published online 02 March 2018

The authors regret to inform the readers that wrong selections of the relative insets may have occurred for Fig. 4g GAPDH bands. In order to reduce the misunderstanding that may be caused in the future, we replaced the images with the alternative ones. It is now correct in the below pictures.

The authors would like to apologise for any inconvenience caused.