# Supplementary material for: Indoleamine 2,3-dioxygenase expression regulates the survival and proliferation of Fusobacterium nucleatum in THP-1-derived macrophages
Source: Cell Death Dis. 2018 Mar 2;9(3):355. doi: 10.1038/s41419-018-0389-0 (PMC5834448; doi:10.1038/s41419-018-0389-0)
Supplement: Supplementary file 1 — Supporting information [file 41419_2018_389_MOESM1_ESM.pdf]

Fig S1: The specific immunofluorescence staining of *Fn* bacteria. *Fn* were observed by confocal microscope (60×) by using mouse and human *Fn* polyclonal primary antibody respectively.

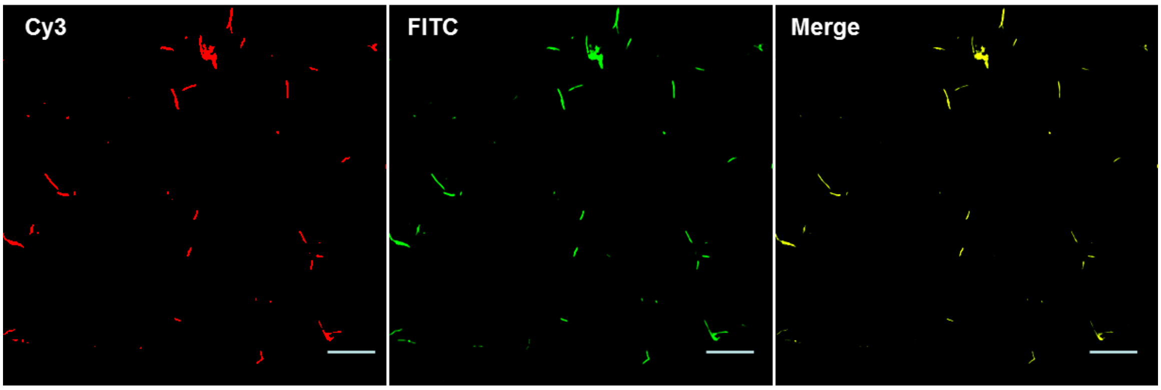

S1 Table: Levels of cytokines in supernatants of THP-1 derived macrophages infected with *F. nucleatum* or Heat-killed *F. nucleatum*

	Mean cytokine concn (pg/ml)±SD							
	Fn				Heat-killed Fn			
Cytokine	0 h	24 h	48 h	72 h	0 h	24 h	48 h	72 h
IL-2	ND	ND	ND	ND	ND	ND	ND	ND
IL-4	ND	ND	ND	ND	ND	ND	ND	ND
IL-6	ND	127.89±16.21*	3717.37±112.42*	4256.35±135.63*	ND	121.5±13.12*	1024.27±28.39*	865.43±19.64*
IL-10	ND	ND	ND	ND	ND	ND	ND	ND
TNF-α	ND	1043.78±53.23*	800.14±35.31*	724.25±34.52*	ND	815.38±21.24*	595.56±18.32*	420.32±12.36*
IFN-γ	ND	ND	ND	ND	ND	ND	ND	ND

ND, not detected. Asterisks indicate a *P* value of <0.001.

S2 Table: Primer sequences used in real-time RT-PCR analysis

Gene	Primer sequence
IDO	F:5’- GGTGGTGGCTCTCCTTGTCATT -3’
	R:5’- GCTTTAAAGTTCCGCACGTCCTT -3’
CCR-7	F:5’- GGTGGTGGCTCTCCTTGTCATT -3’
	R:5’- GCTTTAAAGTTCCGCACGTCCTT -3’
IL-6	F:5’- CCTCCAGAACAGATTTGAGAGTAGT -3’
	R:5’- GGGTCAGGGGTGGTTATTGC -3’
IL-12p40	F:5’- GTTTTATGACAATCAGGAAGAATGC -3’
	R:5’- TTATCAACACCATCTCCAGGAAGTC -3’
IL-12p35	F:5’-CCTTGTGGCTACCCTGGTCCTC-3’
	R:5’- CTCAGCAGGTTTTGGGAGTGGT-3’
TNF-α	F: 5’-GTGCTTGTTCCTCAGCCTCTT-3’
	R: 5’-ATGGGCTACAGGCTTGTCACT-3’
IL-10	F: 5’-ACCTGCCTAACATGCTTCGAG-3’
	R: 5’-CTGGGTCTTGGTTCTCAGCTT-3’
CD206	F:5’-TCTGGTGAACGGAATGATTGTGTA-3’
	R:5’-TCCTTGTGTCAGCTTTTGTTGTAAG-3’
CD80	F:5’- GGGAAAGTGTACGCCCTGTA-3’
	R:5’- GCTACTTCTGTGCCCACCAT -3’
CD163	F:5’- CTGAGCCTGAGACTGGTAGATGG -3’
	R:5’- ATCCCTTACTGGCGTTAACTCGA -3’
CXCR-4	F:5’-CCACCATCTACTCCATCATCTTCTT-3’
	R:5’-TACTTGTCCGTCATGCTTCTCAG -3’
IFN-γ	F:5’- AGAGTGTGGAGACCATCAAGGAAGA -3’
	R:5’- TGCGTTGGACATTCAAGTCAGTTAC -3’
HLA-DR	F:5’- GGCGGCTTGAAGAATTTGGAC-3’
	R:5’- CATTGGTGATCGGAGTATAGTTGGA -3’
IL-1β	F:5’- TCCAGGGACAGGATATGGAG -3’
	R:5’- TCTTTCAACACGCAGGACAG -3’
IL-17	F:5’- TGTCACTGCTACTGCTGCT -3’
	R:5’- GGTTATGGATGTTCAGGTTG -3’
18s RNA	F:5′- AGGTCTGTGATGCCCTTAGATGTC-3′
	R:5′-TCCTCGTTCATGGGGAATAATT-3′
